# Mesangial cells are key contributors to the fibrotic damage seen in the lupus nephritis glomerulus

**DOI:** 10.1186/s12950-019-0227-x

**Published:** 2019-11-14

**Authors:** Rachael D. Wright, Paraskevi Dimou, Sarah J. Northey, Michael W. Beresford

**Affiliations:** 1 Department of Women’s and Children’s Health, Institute of Translational Medicine, University of Liverpool, member of Liverpool Health Partners, Liverpool, UK; 2 Department of Women and Children’s Health, Institute in the Park, Alder Hey Children’s NHS Foundation Trust, Eaton Road, Liverpool, L12 2AP UK; 3 Department of Paediatric Rheumatology, Alder Hey Children’s NHS Foundation Trust, member of Liverpool Health Partners, Liverpool, UK

**Keywords:** Lupus nephritis, Mesangial cells, Fibrosis

## Abstract

**Background:**

Lupus nephritis (LN) affects up to 80% of juvenile-onset systemic lupus erythematosus patients. Mesangial cells (MCs) comprise a third of the glomerular cells and are key contributors to fibrotic changes within the kidney. This project aims to identify the roles of MCs in an in vitro model of LN.

**Methods:**

Conditionally immortalised MCs were treated with pro-inflammatory cytokines or with patient sera in an in vitro model of LN and assessed for their roles in inflammation and fibrosis.

**Results:**

MCs were shown to produce pro-inflammatory cytokines in response to a model of the inflammatory environment in LN. Further the cells expressed increased levels of mRNA for extracellular matrix (ECM) proteins (*COL1A1, COL1A2, COL4A1* and *LAMB1*), matrix metalloproteinase enzymes (*MMP9*) and tissue inhibitors of matrix metalloproteinases (*TIMP1*). Treatment of MCs with serum from patients with active LN was able to induce a similar, albeit milder phenotype. Treatment of MCs with cytokines or patient sera was able to induce secretion of TGF-β1, a known inducer of fibrotic changes. Inhibition of TGF-β1 actions through SB-431542 (an activin A receptor type II-like kinase (ALK5) inhibitor) was able to reduce these responses suggesting that the release of TGF-β1 plays a role in these changes.

**Conclusions:**

MCs contribute to the inflammatory environment in LN by producing cytokines involved in leukocyte recruitment, activation and maturation. Further the cells remodel the ECM via protein deposition and enzymatic degradation. This occurs through the actions of TGF-β1 on its receptor, ALK5. This may represent a potential therapeutic target for treatment of LN-associated fibrosis.

## Introduction

Lupus nephritis (LN) is a severe clinical manifestation of Systemic Lupus Erythematosus (SLE). Patients diagnosed in childhood (< 18 years) have a higher prevalence of LN (up to 80%) and a faster rate of damage accrual in the kidney compared to their adult counterparts [1–4]. Flares of LN occur throughout the disease course and each flare increases the risk of permanent damage by increasing damage accrual within the kidney [5]. LN is initiated by binding of autoantibodies to antigens expressed by native kidney cells [6]. Mesangial cells (MCs) express high levels of antigens that are bound by autoantibodies, including Annexin II and α-actinin, and thus are targets for damage in LN [7, 8]. Binding of autoantibodies to MCs leads to rapid internalisation and initiation of an inflammatory response, an early phase marker of glomerulonephritis in NZB/W F1 mice [9]. The response of MCs to autoantibody binding in lupus nephritis has been extensively studied and it has been demonstrated that an immune response is generated. However, the response of MCs to the inflammatory process itself occurring within LN has yet to be fully investigated.

MCs comprise approximately a third of the cell population within the glomerulus. They play important roles in homeostasis by maintaining the structural architecture of the glomerulus, producing and maintaining the mesangial matrix, regulating the filtration surface area and phagocytosing apoptotic cells or immune complexes [10]. MCs are similar to smooth muscle cells but with modified functions. They have the ability to contract which allows them to contribute to maintaining the structural architecture of the glomerulus and regulate the filtration surface area, but they are also involved in the immune response of the glomerulus [11].

In response to damage (both immune complex deposition and cytokine-induced) MCs contribute to fibrotic changes that are occurring within the glomerulus by undergoing hypertrophy and proliferation. This has been demonstrated both in vitro and in vivo, as well as in biopsy samples taken from LN patients [12, 13]. In addition to hyperproliferating, MCs deposit increased extracellular matrix proteins and increase production of matrix metalloproteinases, resulting in glomerular remodelling [14, 15].

This study aimed to delineate the roles of MCs in the pathogenesis of LN using a cytokine-based, in vitro model with cytokines known to be up-regulated in the sera of patients with active LN [16–19]. Further this study attempted to recapitulate this using a more physiological model in which MCs are treated with sera from patients with LN.

## Results

### Mesangial cells contribute to the pro-inflammatory environment in lupus nephritis

As studies have previously demonstrated that MCs are able to secrete pro-inflammatory cytokines in response to injurious stimuli, this was assessed in an in vitro, cytokine-based LN model. Multiplex analysis was used to determine levels of IL-6, IL-8, IL-10, and M-CSF.

**Fig. 1:**
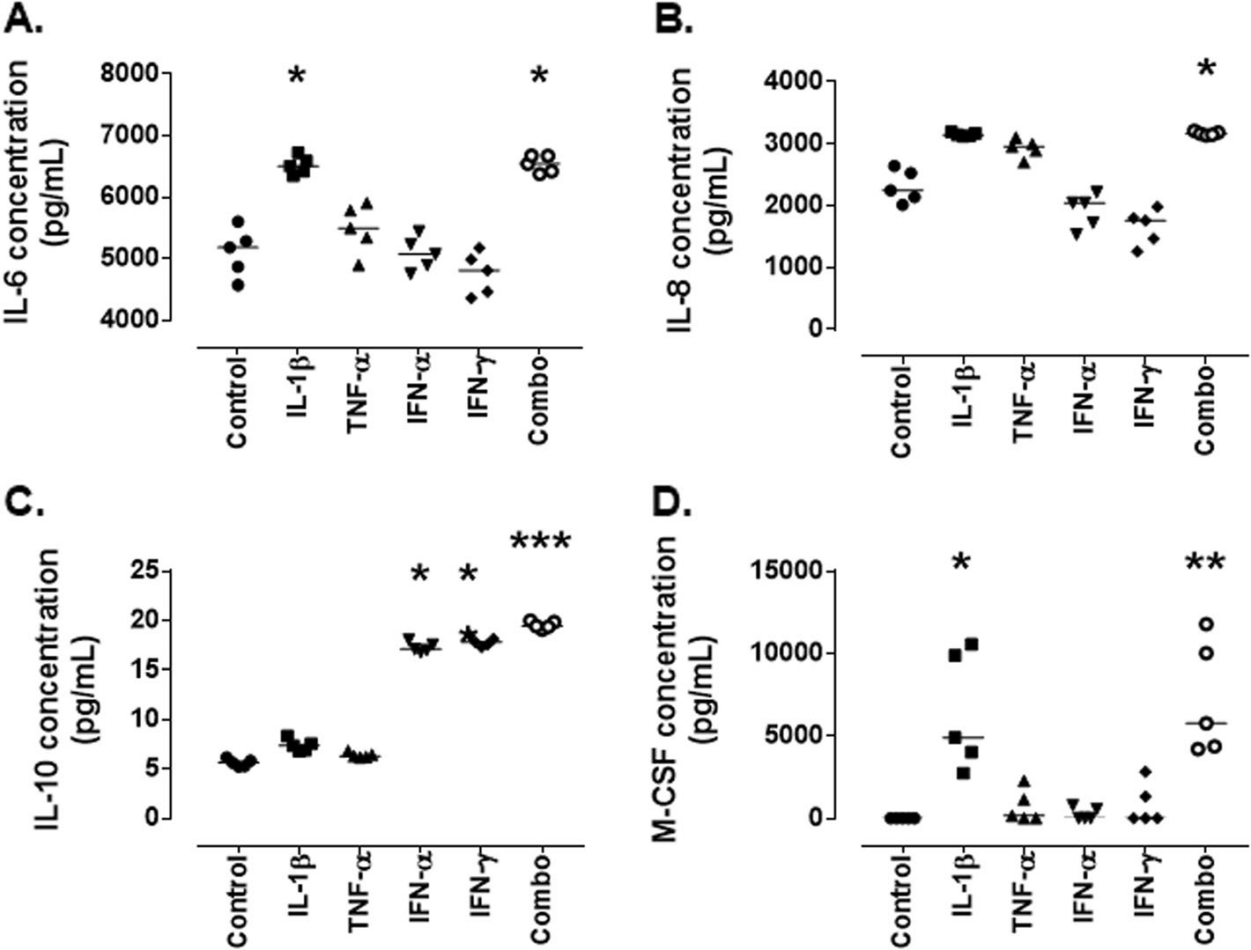
*Cytokine/chemokine expression by conditionally immortalised mesangial cells following cytokine stimulation.* Conditionally immortalised MCs were treated with IL-1β, TNF-α, IFN-α, IFN-γ alone and in combination (Combo) for 24 h. Multiplex was used to assess protein levels of IL-6 (**a**), IL-8 (**b**), IL-10 (**c**) and M-CSF (**d**). *N* = 5 per group, data are analysed using Friedman’s test with Dunn’s post-hoc test, * *P* < 0.05, **P < 0.01 and ***P < 0.001 vs control

Relatively high levels of IL-6 were secreted by untreated MCs (5180 pg/mL [4570-5602]), this was significantly increased in response to treatment with IL-1β (6807 pg/mL [6349-6725]; *p* = 0.03) and the combined treatment (6545 pg/mL [6381-6678]; *p* = 0.02) (Fig. 1a). IL-8 was expressed at high levels under basal conditions (2247 pg/mL [2012-2643]) and this was increased further by treatment with the combination of cytokines (3168 pg/mL [3140-3215]; *p* = 0.03) (Fig. 1b). In contrast, IL-10 was expressed at low levels in the absence of treatment (5.237 pg/mL [5.237–6.18]), but this was significantly increased in response to IFN-α (17.08 pg/mL [16.78–18.05]; *p* = 0.02), IFN-γ (17.81 pg/mL [17.31–18.5]; *p* = 0.001) and the combined cytokine treatment (19.44 pg/mL [19.08–20]; *p* < 0.0001) (Fig. 1c). Baseline expression of M-CSF was below the level of detection for the assay (and were thus set at (minimum detection level/√2): 363.45 pg/mL); however, in response to IL-1β treatment (4905 pg/mL [2739–10,545]; *p* = 0.02] and the combination of cytokines treatment (5781 pg/mL [4218-11,778]; *p* = 0.004) this significantly increased (Fig. 1d).

An attempt was made to recapitulate this effect in a more physiological model of LN using sera from patients with active disease (renal BILAG A/B), inactive disease (renal BILAG D/E) and age- and sex-matched HCs.

**Fig. 2:**
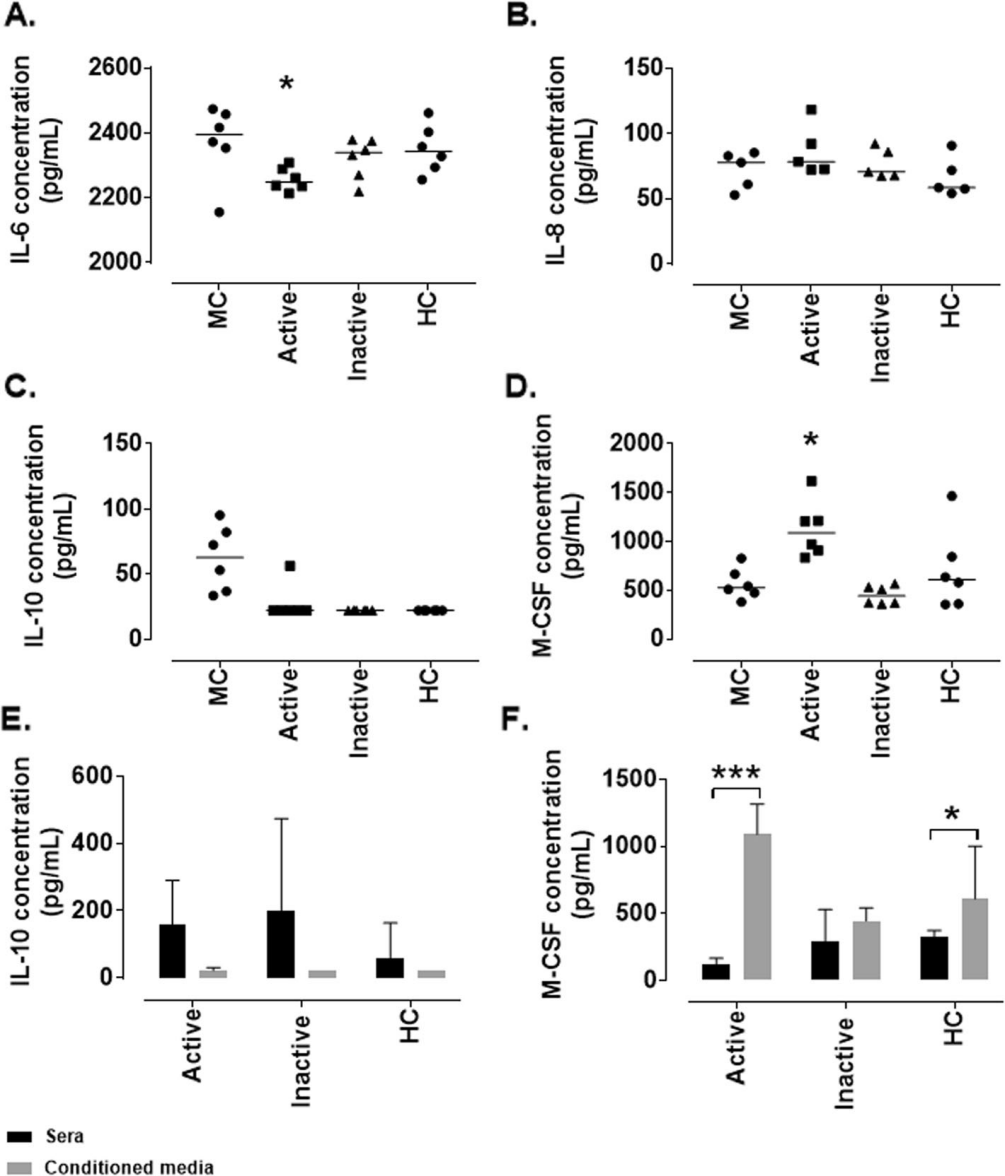
*Cytokine/chemokine expression by conditionally immortalised mesangial cells following stimulation with lupus nephritis patient sera.* Conditionally immortalised MCs were treated with 10% sera from patients with active (rBILAG A/B) and inactive (rBILAG D/E) LN and with age-and sex-matched HCs for 24 h. ELISA was used to assess protein levels of IL-6 in conditioned media (**a**) IL-8 in conditioned media (**b**), IL-10 in conditioned media (**c**), and M-CSF in conditioned media (**d**). IL-10 levels in sera (black bar) and conditioned media (grey bar) (**e**), and M-CSF levels in sera (black bar) and conditioned media (grey bar) (**f**). *N* = 5–6 per group, data are analysed using Kruskal-Wallis test with Dunn’s post hoc test, *P < 0.05 and ***P < 0.001 vs untreated MCs

IL-6 was expressed at relatively high levels in untreated MCs (2396 pg/mL [2156-2475]) and this was significantly reduced following treatment with active LN sera (2249 pg/mL [2214-2308]; *p* = 0.03) (Fig. 2a). IL-8, however, was expressed at baseline but not affected by treatment (Fig. 2b). IL-10 is expressed by untreated MCs (62.83 pg/mL [33.61–95.29]) and following all treatments (except 1 active disease patient sera) this was reduced to below the level of detection for the assay (Fig. 2c). M-CSF was expressed by unstimulated MCs (528.1 pg/mL [381.6–829]) and this was significantly increased in response to treatment with sera from active disease patients (1090 pg/mL [835.2–1616]; *p* = 0.04) (Fig. 2d).

In order to exclude the sera as the source of the cytokines the levels of each cytokine were assessed in RPMI + 10% sera (to mimic that which was used to treat the cells); IL-6 and IL-8 levels in RPMI + 10% sera were below the level of detection for all groups (data not shown). IL-10 was expressed in patient sera, in all groups this was reduced to below the level of significance following treatment of MCs (Fig. 2e). M-CSF on the other hand was relatively lowly expressed in active disease patient sera (121.7 pg/mL [92.49–192.3]) and this was significantly increased in MC conditioned media following treatment (1090 pg/mL [835.2–1616]; *p* < 0.0001). Further M-CSF was relatively lowly expressed in healthy control (HC) sera (320.7 pg/mL [114.3–376.6]) and this was significantly increased in conditioned media following 24 h treatment (608.4 pg/mL [356.7–1467]; *P* = 0.016) (Fig. 2f).

### The lupus nephritis pro-inflammatory environment contributes to extracellular matrix remodelling by the mesangial cells

**Fig. 3:**
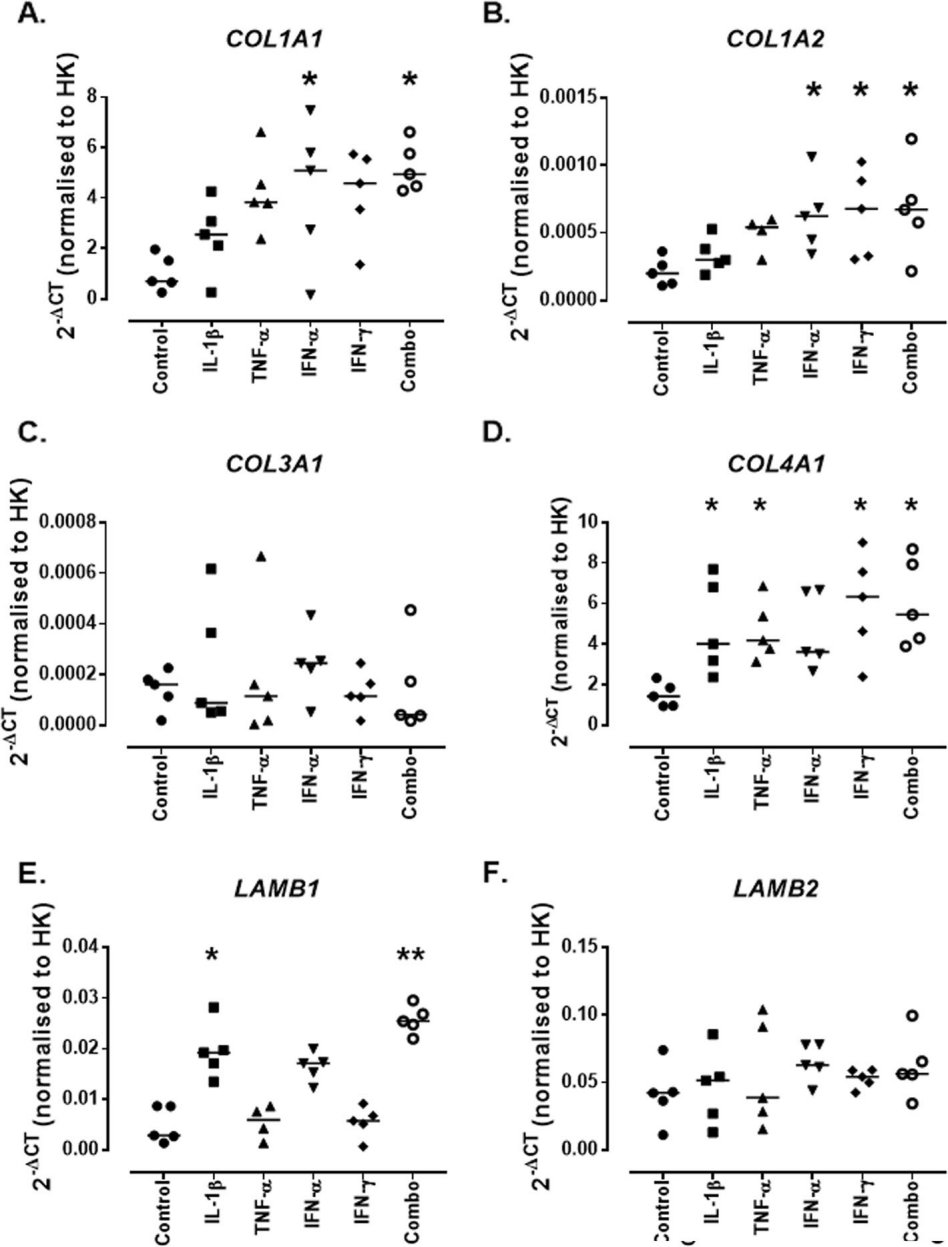
*Expression of mRNA for ECM genes by conditionally immortalised mesangial cells following cytokine stimulation.* Conditionally immortalised MCs were treated with IL-1β, TNF-α, IFN-α and IFN-γ alone and in combination (Combo) for 24 h. mRNA expression was assessed for *COL1A1* (**a**)*, COL1A2* (**b**)*, COL3A1* (**c**)*, COL4A1* (**d**)*, LAMB1* (**e**) and *LAMB2* (**f**). *N* = 5 per group, data are analysed using Friedman’s test with Dunn’s post-hoc test, *P < 0.05 and **P < 0.01 vs control

MCs are heavily involved in maintenance of the ECM and in response to damage secrete proteins and enzymes that restructure the matrix. The expression of genes involved in remodelling the ECM were assessed in response to 24 h cytokine treatments. MCs expressed low levels of *COL1A1* mRNA at baseline (0.708 [0.262–1.96]) and this was significantly increased in response to treatment with IFN-γ (5.089 [0.169–7.484]; *p* = 0.03) and the combination of cytokines (4.951 [4.299–6.628]; *p* = 0.03) (Fig. 3a). Relatively low levels of mRNA for *COL1A2* were expressed by untreated MCs (0.0002 [0.0001–0.0003]), this was significantly increased in response to IFN-α (0.0006 [0.0003–0.001]; *p* = 0.03), IFN-γ (0.0006 [0.0003–0.001]; *p* = 0.04) and the combination of cytokines (0.006 [0.0002–0.001]; *p* = 0.03) (Fig. 3b). *COL4A1* mRNA was expressed at low levels in control MCs (1.428 [0.945–2.335]), this was significantly increased by treatment with IL-1β (4.021 [2.375–7.703]; *p* = 0.05), TNF-α (4.195 [3.144–6.859]; *p* = 0.03), IFN-γ (6.331 [2.398–9.013]; *p* = 0.03) and the combination of cytokines (5.453 [3.908–8.688]; *p* = 0.02) (Fig. 3d). MCs expressed low levels of *LAMB1* mRNA under baseline conditions (0.002 [0.001–0.008]), this was significantly increased in response to treatment with IL-1β (0.019 [0.013–0.028]; *p* = 0.05) and the combination of cytokines (0.025 [0.022–0.029]; *p* = 0.002) (Fig. 3e). MCs expressed mRNA for *COL3A1* and *LAMB2* however these were not affected by treatment (Fig. 3c and e).

**Fig. 4:**
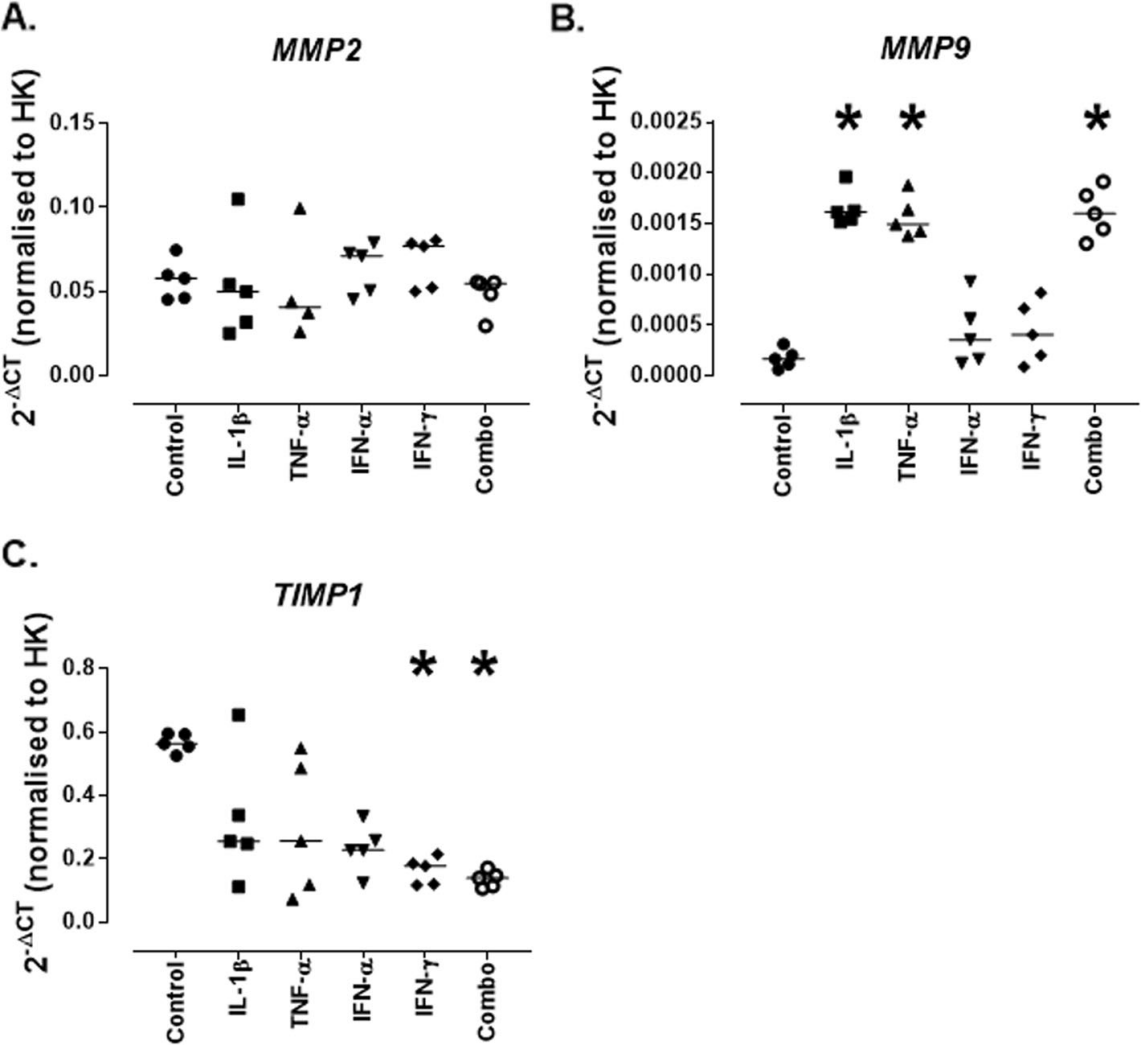
*Expression of mRNA for remodelling enzymes genes by conditionally immortalised mesangial cells following cytokine stimulation.* Conditionally immortalised MCs were treated with IL-1β, TNF-α, IFN-α and IFN-γ alone and in combination (Combo) for 24 h. mRNA expression was assessed for *MMP2* (**a**)*, MMP9* (**b**)*,* and *TIMP1* (**c**). *N* = 5 per group, data are analysed using Friedman’s test with Dunn’s post-hoc test, *P < 0.05 vs control

In order to obtain a clear representation of the remodelling of the ECM it is important to assess the expression of ECM protein genes, MMPs and TIMPs. MCs have been shown to express MMP2 and MMP9, as well as TIMP1 [20, 21] and thus these were assessed following cytokine stimulation. Relatively low levels of *MMP9* mRNA were expressed by untreated MCs (0.0001 [0.00006–0.0003]), this was significantly increased in response to treatment with IL-1β (0.0016 [0.0015–0.0019]; *p* = 0.01), TNF-α (0.0015 [0.0014–0.0019]; *p* = 0.02) and the combination of cytokines (0.0016 [0.0013–0.0019]; *p* = 0.03) (Fig. 4a). *TIMP1* was expressed at relatively high levels in control MCs (0.564 [0.526–0.595]), this was significantly decreased in response to IFN-γ (0.178 [0.116–0.215]; *p* = 0.01) and the combination of cytokines (0.139 [0.106–0.172]; *p* = 0.01) (Fig. 4c). *MMP2* mRNA was also expressed by MCs but was not significantly affected by any of the treatments (Fig. 4a).

**Fig. 5:**
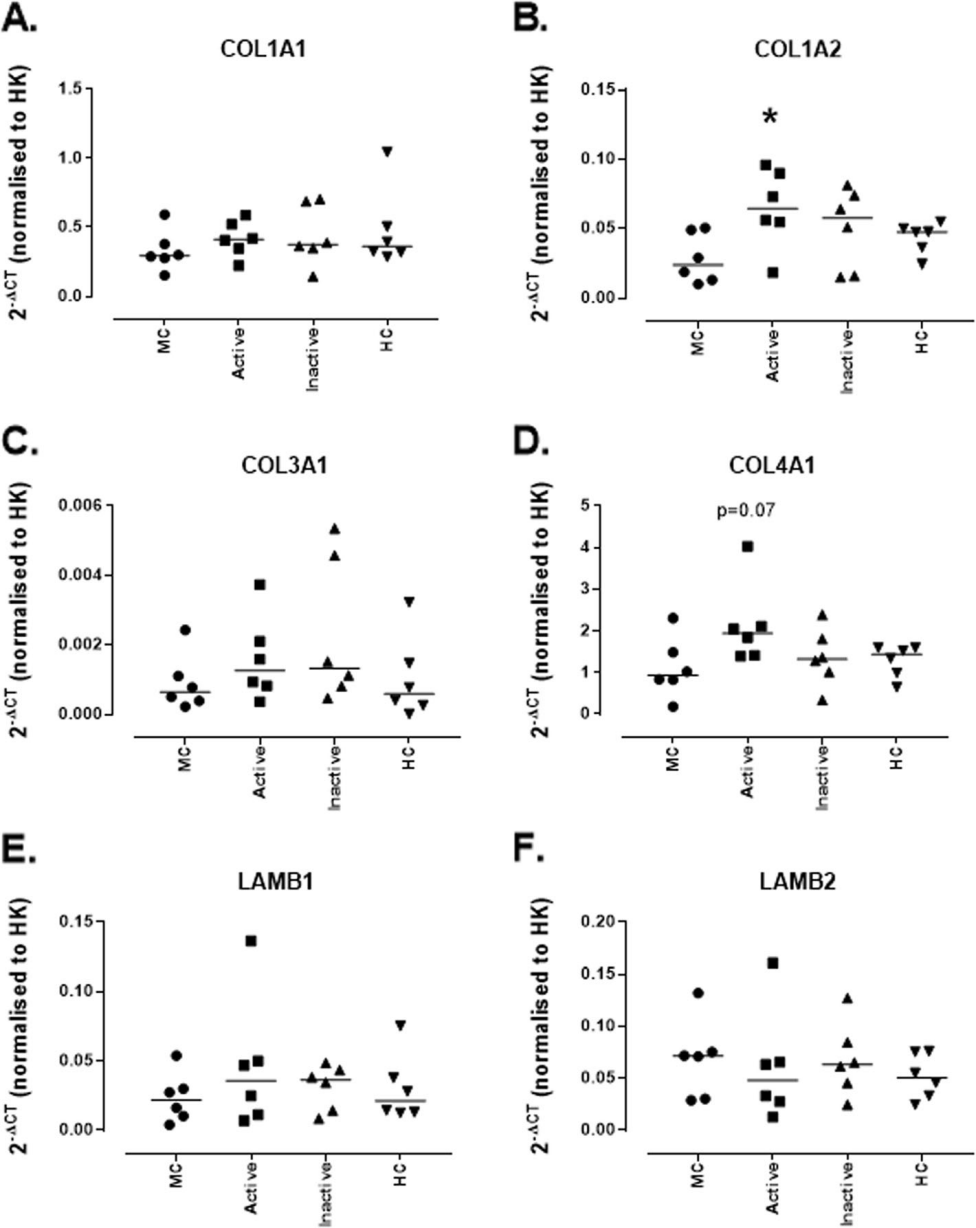
*Expression of mRNA for ECM genes by conditionally immortalised mesangial cells following stimulation with lupus nephritis patient sera.* Conditionally immortalised MCs were treated with 10% sera from patients with active (rBILAG A/B) and inactive (rBILAG D/E) LN and with age-and sex-matched HCs for 24 h. mRNA expression was assessed for *COL1A1* (**a**)*, COL1A2* (**b**)*, COL3A1* (**c**)*, COL4A1* (**d**)*, LAMB1* (**e**) and *LAMB2* (**f**). *N* = 5–6 per group, data are analysed using Kruskal-Wallis test with Dunn’s post-hoc test, *P < 0.05 vs untreated MCs

This was recapitulated in the more physiological model of treating with RPMI (+ 10% patient sera). MCs expressed mRNA for *COL1A1* and *COL3A1* under normal conditions and these were not significantly modulated following treatment with 10% LN patient sera (Fig. 5a and c). Prior to treatment *COL1A2* mRNA was expressed at relatively low levels (0.00065 [0.00022–0.0024]), this was significantly increased in response to treatment with active sera (0.0012 [0.0003–0.003]; *p* = 0.03) while inactive and HC sera had no effect (Fig. 5b). *COL4A1* mRNA was expressed by untreated MCs (0.933 [0.181–2.307]), a trend was seen towards an increase with active sera (1.947 [1.397–4.028]; *p* = 0.07) however this did not reach significance. No other treatments induced a change in *COL4A1* mRNA (Fig. 5d). MCs express mRNA for *LAMB1* and *LAMB2* however these were not affected by any of the sera treatments (Figs. 5e-f).

**Fig. 6:**
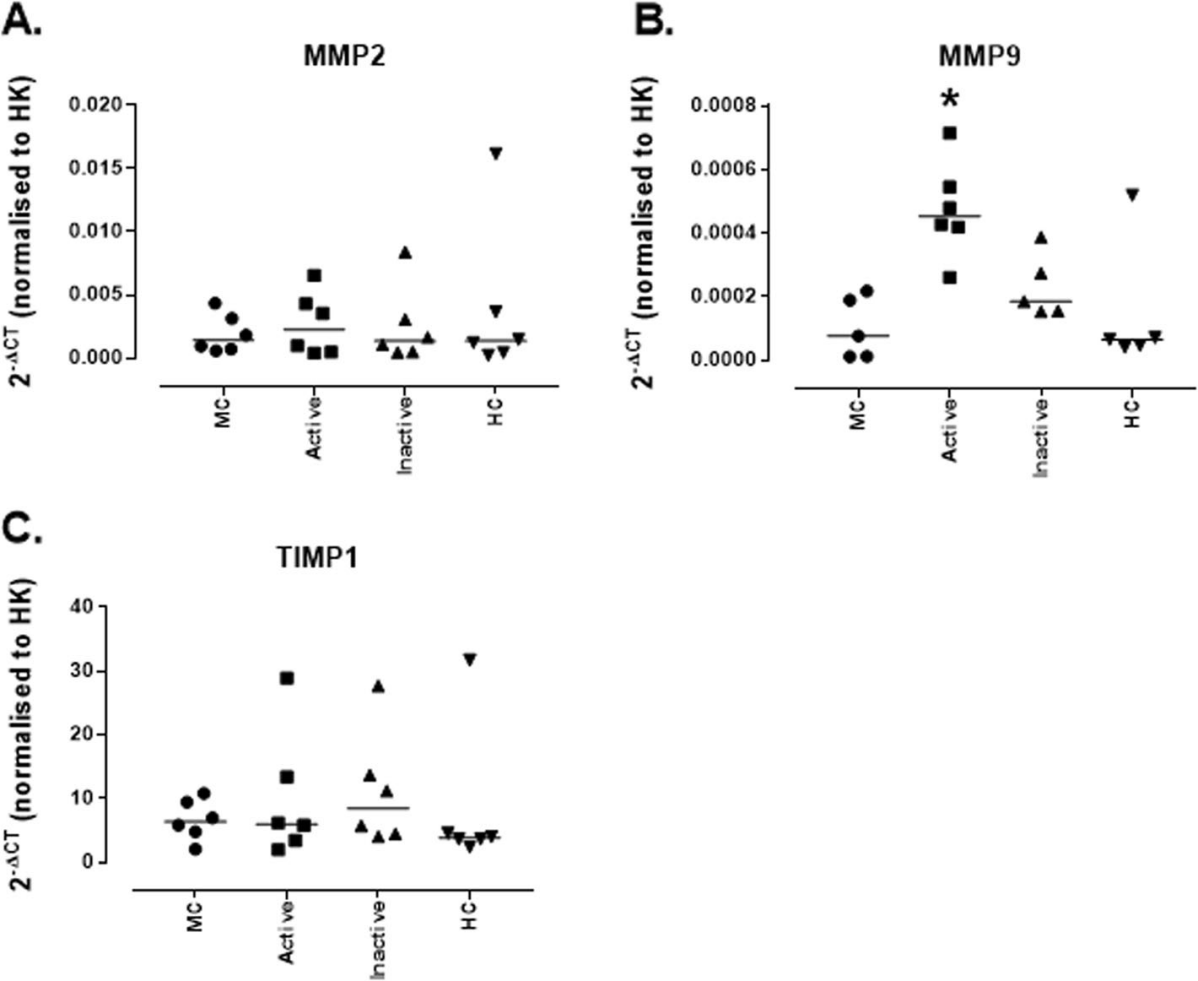
*Expression of mRNA for remodelling enzymes genes by conditionally immortalised mesangial cells following stimulation with lupus nephritis patient sera.* Conditionally immortalised MCs were treated with 10% sera from patients with active (rBILAG A/B) and inactive (rBILAG D/E) LN and with age-and sex-matched HCs for 24 h. mRNA expression was assessed for *MMP2* (**a**)*, MMP9* (**b**)*,* and *TIMP1* (**c**). *N* = 5–6 per group, data are analysed using Kruskal-Wallis test with Dunn’s post-hoc test, *P < 0.05 vs untreated MCs

Levels of mRNA for remodelling enzymes were also assessed. *MMP2* and *TIMP1* mRNA were expressed by untreated MCs but levels were not affected by any of the sera treatments (Fig. 6a and c). *MMP9* mRNA was expressed by MCs under normal conditions (0.000078 [0.000011–0.00022]) and this was significantly increased by treatment with sera from active LN patients (0.00045 [0.00026–0.00071]; *p* = 0.011), no other treatments induced any changes in *MMP9* mRNA (Fig. 6b).

### Extracellular matrix remodelling by mesangial cells is induced by TGF-β1

**Fig. 7:**
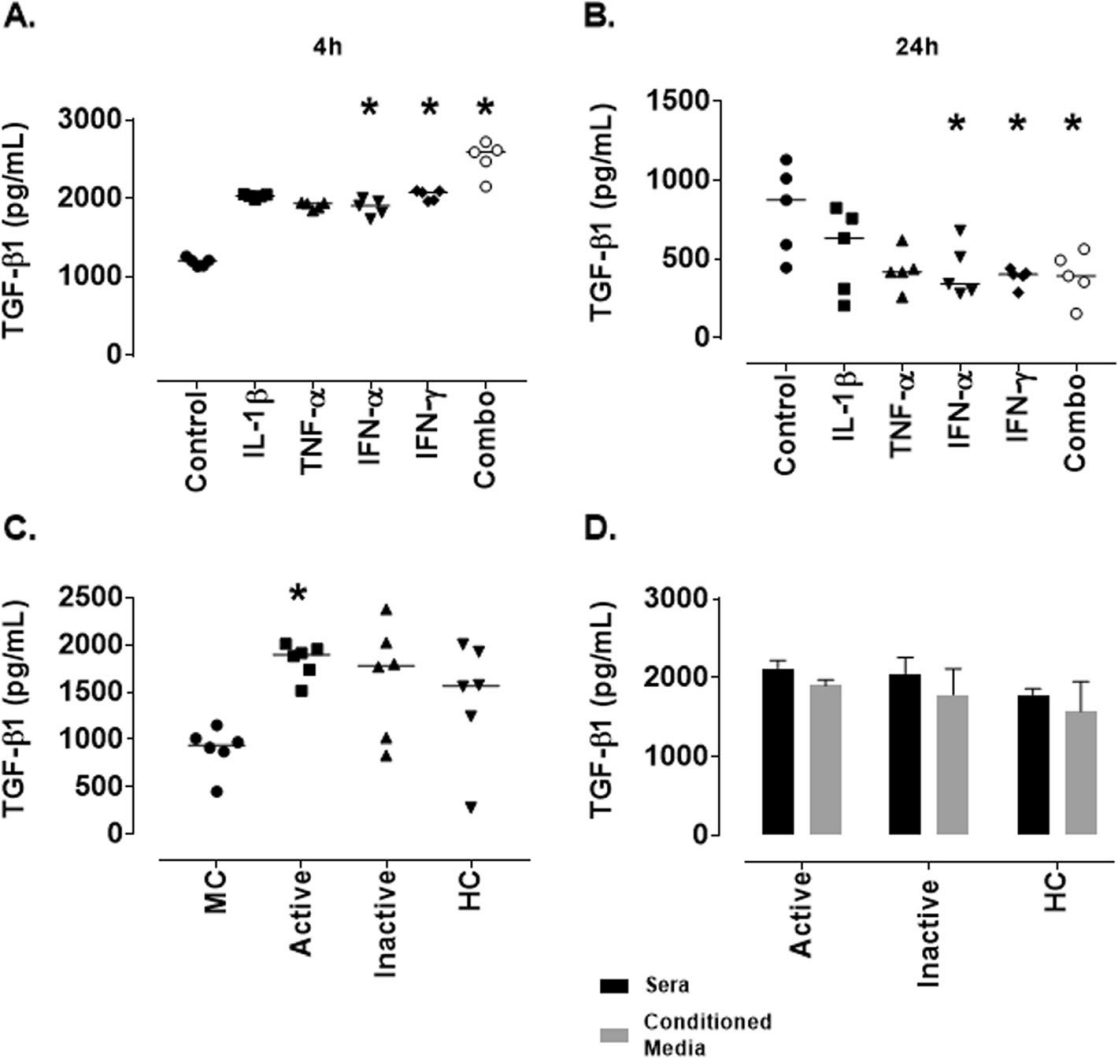
*Production of latent TGF-β1 by conditionally immortalised mesangial cells following stimulation with cytokines or lupus nephritis patient sera.* Conditionally immortalised MCs were treated with IL-1β, TNF-α, IFN-α and IFN-γ alone and in combination (Combo) for 4 and 24 h. Or with 10% sera from patients with active (rBILAG A/B) and inactive (rBILAG D/E) LN and with age-and sex-matched HCs for 24 h. Levels of latent TGF-β1 were assessed in conditioned media from 4 h cytokine treatments (**a**), 24 h cytokine treatments (**b**), 24 h sera treatments (**c**) and directly in the sera (black bar) compared to conditioned media from sera treatments (grey bar) (**d**). *N* = 5–6 per group, data are analysed using Friedman’s test (cytokine treatments) or Kruskal-Wallis test (sera treatments) with Dunn’s post-hoc test, *P < 0.05 vs control (**a**,**b**) or MC (**c**)

One of the most common mediators of ECM remodelling is transforming growth factor (TGF)-β1 therefore the expression of latent TGF-β1 following cytokine stimulation was assessed in our model. As we were keen to investigate the role of TGF-β1 in inducing the changes seen at 24 h the levels were assessed at 4 h and 24 h to look at temporal modulation. At 4 h post-cytokine stimulation latent TGF-β1 was expressed at relatively high levels (1201 pg/mL [1129-1257], this was significantly increased following treatment with IL-1β (2028 pg/mL [1984-2051]; *p* = 0.02), IFN-γ (2,076 pg/mL [1963-2096]; *p* = 0.02) and the combination of cytokines (2589 pg/mL [2152-2721]; *p* = 0.0001) (Fig. 7a). At 24 h however, latent TGF-β1 was expressed by untreated MCs (873.5 pg/mL [443.4–1130], but this was decreased following treatment with IFN-α (340 pg/mL [280.4–679.8]; *p* = 0.05), IFN-γ (400.7 pg/mL [285.2–438]; *p* = 0.02) and the combination of cytokines (391.3 pg/mL [151.5–561.7]; *p* = 0.03) (Fig. 7b).

As MCs treated with patient sera appeared to have a milder phenotype than those treated with cytokines the levels of latent TGF-β1 were assessed only at 24 h. When MCs were treated with patient sera for 24 h there was a significant increase in the production of latent TGF-β1 by MCs treated with active sera (1897 pg/mL [1515-2010]; *p* = 0.014) compared to untreated MCs (941.1 pg/mL [449.9–1150]) while no other treatments had a significant effect (Fig. 7c). The expression of latent TGF-β1 in RPMI (+ 10% patient sera) was also assessed and it was determined that although no significant difference in the expression was seen between the groups this was similar to that seen in conditioned media following MC stimulation (Fig. 7d).

**Fig. 8:**
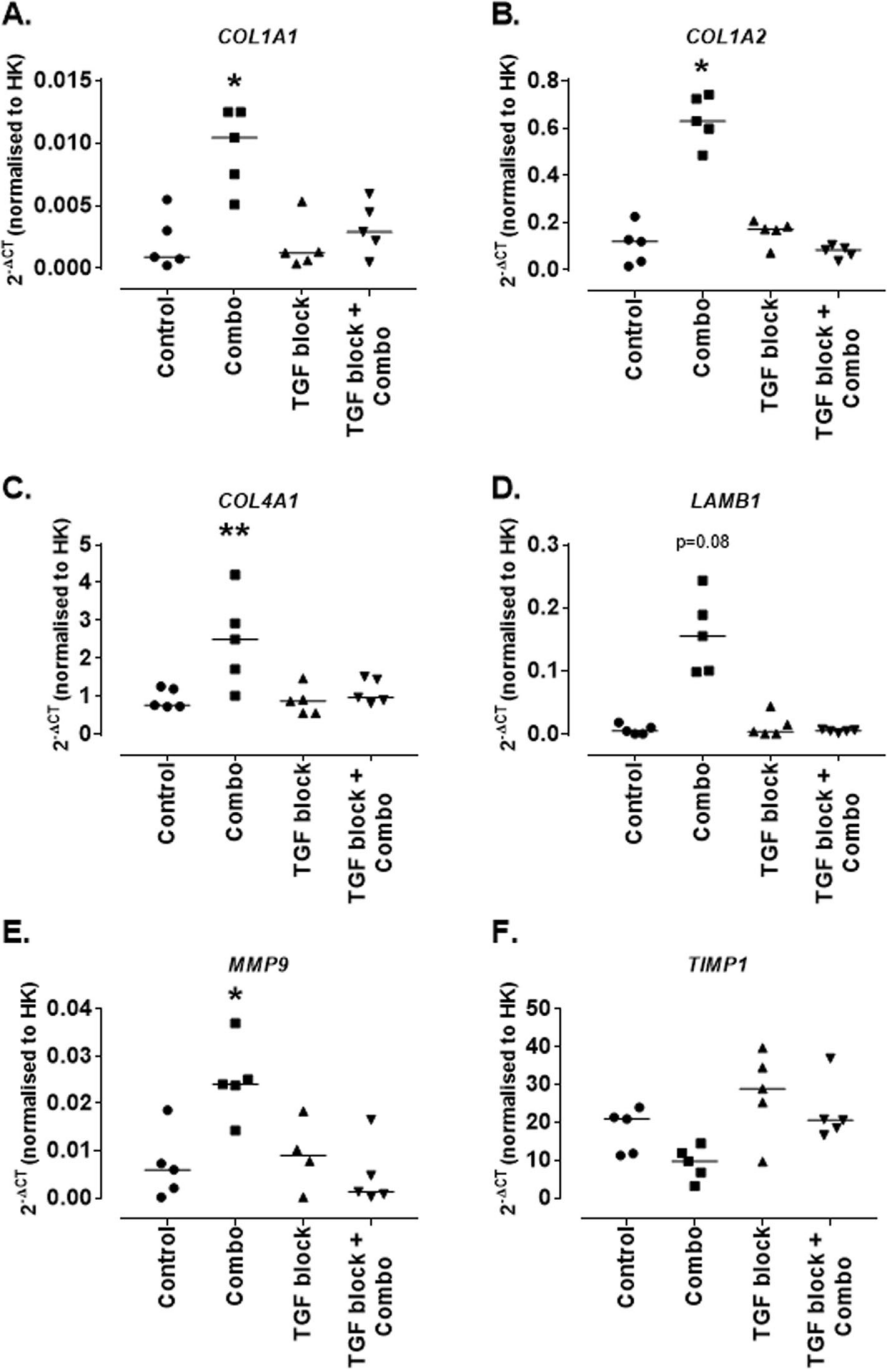
*Expression of mRNA for ECM genes by conditionally immortalised mesangial cells following cytokine stimulation with TGF-β1 receptor blockade.* Conditionally immortalised MCs were treated with SB-431542 for 30 mins before stimulation with a combination of TNF-α, IL-1β, IFN-α and IFN-γ. mRNA expression was assessed for *COL1A1* (**a**)*, COL1A2* (**b**)*, COL4A1* (**c**)*, LAMB1* (**d**)*, MMP9* (**e**) and *TIMP1* (**f**). *N* = 5 per group, data are analysed using Friedman’s test with Dunn’s post-hoc test, *P < 0.05 and **P < 0.01 vs untreated MCs

TGF-β1 receptor activity blockade was induced by pre-treatment with SB-431542 and following this MCs were treated with the combination of cytokines treatment (IL-1β, TNF-α, IFN-α and IFN-γ) for 24 h as previously and the expression of mRNA for ECM genes was assessed. As demonstrated previously the combination of cytokines induced increased expression of mRNA for *COL1A1, COL1A2, COL4A1, LAMB1, MMP9* and *TIMP1*. MCs express mRNA for *COL1A1* at baseline (0.0009 [0.0002–0.005]) and this is significantly increased following stimulation with the combination of cytokines (0.01 [0.005–0.012]; *p* = 0.02). Pre-treatment with SB-431542 was able to reduce this close to baseline levels (0.002 [0.0005–0.005]) (Fig. 8a). *COL1A2* mRNA is also expressed by MCs at baseline (0.1213 [0.0168–0.2257]) and again this is significantly increased in response to the combination of cytokines (0.6297 [0.4857–0.7428]; *p* = 0.02), again this was reduced to similar to baseline levels by pre-treatment with SB-431542 (0.0858 [0.0387–0.1071]) (Fig. 8b). Untreated MCs express mRNA for *COL4A1* (0.7654 [0.7259–1.255]) and this is significantly increased in response to the combination of cytokines (2.501 [1.016–4.212]; *p* = 0.009), this was unchanged compared to control following pre-treatment with SB-431542 (0.9711 [0.8184–1.515]) (Fig. 8c). *LAMB1* mRNA is expressed by untreated MCs (0.004 [0.0003–0.018]) and trended towards an increase following treatment with the combination of cytokines (0.1551 [0.098–0.243]; *p* = 0.08), levels returned to baseline following pre-treatment with SB-431542 (0.005 [0.0015–0.0076]) (Fig. 8d). The expression of mRNA for *MMP9* was also assessed – this was at relatively low levels at baseline (0.005 [0.0001–0.018]) and was significantly increased following stimulation with the combination of cytokines (0.024 [0.014–0.0369]; *p* = 0.047) and again, this was reduced following pre-treatment with SB-431542 (0.001 [0.0002–0.0165]) (Fig. 8e). Finally, the expression of mRNA for *TIMP1* was assessed – this was expressed by untreated MCs (20.84 [11.3–23.93]). Although it appears that a decrease in expression can be seen following treatment with the combination of cytokines this did not reach significance (9.754 [3.178–14.41]; *p* = 0.6). Following pre-treatment with SB-431542 the levels appeared closer to that seen in the untreated cells (20.47 [16.54–36.94]) (Fig. 8f).

## Discussion

LN is a severe manifestation of juvenile-onset SLE and is, alongside infection and cardiovascular disease, a major cause of SLE-associated morbidity and mortality in these children [3]. MCs are important contributors to the fibrotic damage seen in glomerular diseases as they respond to injurious stimuli by proliferating and producing increased levels of ECM proteins [12–15]. Delineating the path by which these changes occur may unveil new avenues for investigation in LN therapy.

To mimic the inflammatory environment of the LN kidney in vitro a model was designed using key cytokines known to be upregulated in the sera of patients with active LN compared to inactive disease and HCs [16–19].

The contribution of MCs to the inflammatory milieu within the kidney was investigated by assessing the levels of pro-inflammatory cytokines and chemokines produced by MCs in response to the in vitro model. This study demonstrated that MCs express IL-6, IL-8 and IL-10 under normal conditions. This correlates with previously published data that demonstrated that IL-10 is essential for maintaining homeostasis within the kidney [22] and low levels of IL-6 and IL-8 are expressed by MCs in vitro [23, 24]. In response to the cytokines in our model, the levels of all three cytokines significantly increased. High levels of IL-6, IL-8 and IL-10 are important for the recruitment and maturation of neutrophils, B cells and T cells to the glomerulus [25]. In this model, M-CSF was not expressed/very lowly expressed by untreated MCs but was released at high levels following inflammatory stimulation. M-CSF is involved in the recruitment and maturation of macrophages [25]. It is important to note that a previous study in murine primary cells demonstrated IL-6 and IL-8 levels of approximately 80 pg/mL and 75 pg/mL respectively [24]. However, this discrepancy with our findings can be explained by the different cell type analysed. These data demonstrate that in response to inflammatory activation, MCs are potentially playing an important contributory role in the recruitment and activation of immune cells from the circulation, and thus exacerbating the inflammatory response seen.

Further, a more physiological model was used in which sera from patients with LN were used to stimulate MCs and the cytokine levels assessed. This demonstrated that serum treatments were unable to recapitulate the increase in IL-8 seen following cytokine stimulation. Active serum was able to induce an increase in M-CSF secretion compared to untreated MCs while no other treatments had an effect suggesting that something within the active sera was able to induce this secretion that was not present in the other groups. M-CSF levels in the sera were not different between groups. IL-6 and IL-10 were reduced in response to serum treatments, IL-6 levels were below the level of detection for the assay in all sera tested so it is unclear how these may differ between groups. IL-10, however, did not differ between patient groups and all groups reduced MC secretion suggesting that the IL-10 present in the sera may be binding MCs and initiating a negative feedback response, reducing the secretion from MCs.

One of the main known roles of MCs in glomerular injury is the induction of pro-fibrotic changes by remodelling of the ECM. This study identified an increase in the expression of genes responsible for the deposition of ECM proteins in response to the in vitro, cytokine-based model of LN. Increases in *COL1A1*, *COL1A2* and *COL1A4* confirm that enhanced deposition of both healthy (type IV) and pathogenic (type 1) collagens are occurring in this model. This mimics what is seen in lupus-prone mice (NZBWF1/J mice) where increased type 1 collagen is seen to be deposited in the early stages of glomerulonephritis development [26] and in graft-vs-host disease where early deposition of type IV collagen is seen [27]. Increased collagen IV deposition has also been seen in the mesangium in human LN biopsies [28]. Further increased deposition of *LAMB1* is occurring, laminin β1 has been shown to be increased in a model of graft-vs-host disease in early disease but then reduces as disease progresses [29], increased laminin deposition was also seen in two of five human LN biopsies [28]. It is important to note that in the mouse model laminin deposition occurred only early in disease course and it is unclear at what stage of a flare a biopsy would have been taken in the human disease cohort.

When the MCs were treated with LN patient sera there were significant increases noted for active disease sera in collagen I and a trend towards an increase in collagen IV, this suggests that similar changes may be occurring but that these are milder than that seen with cytokines. This may be due to the low concentration of serum used (10%) compared to that seen in blood (45–50%) or potentially due to the immunosuppressant treatment regimens being followed by the patients (Table 1).

**Table 1:** Demographics, renal BILAG scores and medications for LN patients

Demographics	Active LN (*n* = 6)	Inactive LN (*n* = 6)	Healthy Controls (*n* = 6)
Age (years) (median [range])	15.53 [12.18–16.58]	14.55 [11.03–17.79]	14.9 [12.12–16.6]
Age at diagnosis (years) (median [range])	12.8 [6.28–13.29]	10.46 [6.28–16.88]	–
Females (%)	100 (6)	100 (6)	100 (6)
Nationality % (n)			
White British	16.6 (1)	33.3 (2)	100 (6)
Chinese	16.6 (1)	16.6 (1)	0
Somali	33.3 (2)	33.3 (2)	0
African	16.6 (1)	16.6 (1)	0
Indian	16.6 (1)	0	0
Renal BILAG domains			
Renal Hypertension (%) n	16.6 (1)	0	–
Urine ACR (mg/dL) (median [range])	239.3 [0.7–592.4]	7.5 [0.8–8.8]	
Renal Creatinine (mg/dL) (median [range])	45 [37–62]	51 [30–61]	
Estimated GFR (mL/min/1.73m^2^) (median [range])	138 [99.8–158.1]	121.5 [99.1–181.9]	
Medications (n)			
Hydroxychloroquine	4	3	–
Azathioprine	0	3	
Mycophenolate mofetil	6	3	
Prednisolone	5	5	
Methotrexate (oral)	0	0	
Rituximab	1	0	
Cyclophosphamide	1	0	

*ACR* Albumin creatinine ratio, *GFR* Glomerular filtration rate

ECM remodelling is a combination of the increased deposition of proteins and increased enzymatic breakdown of these proteins. Therefore, we looked at the mRNA levels of the main enzymes involved in MC remodelling – MMP2 and MMP9, no significant changes in the expression of MMP2 were noted but MMP9 levels were significantly increased in response to treatment with IL-1β and TNF-α. This correlates with a study that demonstrated that at the onset of proteinuria in lupus-prone (NZBxNZW F1) mice there is an increase in proteolytic activity that can be attributed to MMP9 expression [14]. Further a decrease in the expression of TIMP1 (an MMP inhibitor) was seen with IFN-α and the combination of cytokines treatment suggesting that overall there is a net increase in the activity of these degradation enzymes.

These changes in enzymes were also assessed in MCs following sera treatments and the increase in MMP9 was recapitulated following treatment with active sera while no changes in MMP2 or TIMP1 could be detected.

One of the main drivers of fibrosis is TGF-β1 therefore we looked at levels of TGF-β1 in each of our models to determine whether this could be driving the fibrotic changes we are seeing. At 4 h post-cytokine treatment a significant increase in latent TGF-β1 could be seen and this was decreased at 24 h suggesting the TGF-β1 may be being internalised and eliciting downstream effects. It has previously been shown that in rat mesangial cells TGF-β1 is an autocrine mediator of fibrotic change [30] and thus could explain this temporal modulation. As it was found that the LN patient sera treatments induced a milder phenotype the expression of latent TGF-β1 was only assessed at 24 h where it was shown to be significantly increased in response to treatment with sera from patients with active disease (renal BILAG A/B). This may suggest that the response to sera treatments is delayed compared to that of cytokine treatments. To differentiate between de novo production of TGF-β1 and levels already present in the sera an ELISA was performed to determine the concentration of latent TGF-β1 in RPMI (+ 10% patient sera). Levels were found to be almost identical to that seen in the conditioned media from MCs. When considering that MCs themselves produce approximately 1 ng/mL TGF-β1 this may suggest that a reduction is occurring through internalisation or that a negative feedback loop is occurring due to the expression of TGF-β1 in the sera.

MCs express ALK5 and through this receptor TGF-β1 can induce downstream ECM remodelling [31]. An ALK5 blocker SB-431542 was used to inhibit the effects of TGF-β1 in this model and was able to attenuate all ECM remodelling genes previously shown to be up-regulated in our model following cytokine treatment suggesting that this remodelling is occurring via TGF-β1 activity.

Given that mesangial cells do not show increased expression of these markers in response to human sera we are unable to demonstrate the use of TGF-β1 blockade using SB-431542 in a more physiological model, one future possibility may be to demonstrate that mesangial cells show increased expression of these markers in human kidney tissue, such as from kidney biopsies for patients with lupus nephritis or explore mouse models.

## Conclusion

In conclusion, our cytokine-based in vitro model of LN induced an increase in mediators involved in eliciting an inflammatory response within the glomerulus and further promoted ECM remodelling by increasing the expression of genes involved in protein deposition and enzymatic degradation. Treatment of MCs with patient sera was able to induce a similar, albeit milder phenotype. This could be inhibited by blocking TGF-β1 receptor activity, potentially identifying the inhibition of TGF-β1 activity as a potential future therapeutic target in glomerular fibrotic changes in LN.

## Materials and methods

### Materials

All recombinant cytokines were purchased from Peprotech, London, UK. All primers were purchased from Eurofins Genomics, Ebersberg, Germany.

### Human conditionally immortalised mesangial cell culture

Human conditionally immortalised MCs were kindly provided by Professor Moin Saleem (Children’s Renal Unit and Academic Renal Unit, University of Bristol, Southmead Hospital, Bristol, UK). These cells were conditionally immortalised using the temperature sensitive large T antigen-SV-40 transgene as previously described [32]. These cells have been shown to differentiate fully by 7–10 days after switching from 33 °C to 37 °C. Cell passages between 15 and 30 were used in all experiments, for all experiments *n* = 5–6 independent experiments were used. MCs were routinely cultured in RPMI-1640 medium with L-glutamine (Lonza, Leeds, UK) supplemented with 10% foetal calf serum (ThermoScientific) and insulin transferrin selenium (Sigma-Aldrich, Dorset, UK).

After 7–10 days of differentiation conditionally immortalised MCs were treated with cytokines designed to model the inflammatory environment of the kidney in LN patients, namely: IL-1β, TNF-α, IFN-α and IFN-γ (all known to be involved in the pathogenesis of LN) at 10 ng/mL each alone and in combination (i.e. 10 ng/mL each of IL-1β, TNF-α, IFN-α and IFN-γ altogether). These were chosen as being key cytokines known to be upregulated in the sera of patients with active LN compared to inactive disease and healthy control [16–19]. Following 24 h incubation conditioned media were collected, and RNA was extracted using Trizol (ThermoScientific).

Upon routine clinical visits, patients within the UK JSLE Cohort Study are assessed according to the British Isles Lupus Assessment Group (BILAG) 2004 index [33, 34]. Following differentiation MCs were also treated with 10% sera from patients with active LN (renal BILAG A/B), inactive LN (renal BILAG D/E) and age- and sex- matched HCs (Table 1). Following 24 h incubation conditioned media were collected, and RNA was extracted using Trizol (ThermoScientific).

Cells were pre-treated for 30 mins with SB-431542 (ALK5 receptor blocker) to inhibit TGF-β1 binding as previously described [35] before stimulation with the combined cytokine treatment. Following this conditioned media was collected and RNA was extracted using Trizol.

### Multiplex

A Luminex magnetic bead assay was purchased from R&D Systems, Abingdon UK which was able to detect IL-6, IL-8, IL-10 and M-CSF. The assay was performed on conditioned media collected from cells treated with cytokines for 24 h according to manufacturer’s instructions to assay protein levels in conditioned media from cytokine-treated MCs. The plate was read using a Merck Millipore Luminex MAGPIX® analyser.

### ELISA

TGF-β1, IL-6, IL-8 and M-CSF DuoSets were purchased from R&D Systems, Abingdon, UK. The assays were performed on conditioned media from cells that had been stimulated with cytokines or patient sera for 24 h according to the manufacturer’s instructions to determine protein levels in conditioned media from treated MCs.

### qRT-PCR

RNA was extracted from MCs treated with cytokines for 24 h using the RNeasy miniprep kit (Qiagen, Manchester, UK) following the manufacturer’s instructions. The RNA concentration was determined by Nanodrop and 200-500 ng RNA was transcribed into cDNA using either the AffinityScript multi-temp cDNA synthesis kit (Agilent Technologies, Cheshire, UK) following the manufacturer’s instructions for 24 h cytokine treatments or the Primerdesign all-in-one Reverse Transcription mix (Primerdesign, York, UK) following manufacturer’s instructions (for sera and TGF-β1 blocking assays). qRT-PCR was performed using the primers described in (Table 2) with the Brilliant III Ultra-fast SYBR QPCR mastermix kit (Agilent Technologies) following the manufacturer’s instructions (for 24 h cytokine treatments) or the Primerdesign PrecisionPLUS qPCR Master Mix kit (for sera and TGF-β1 blocking assays). The geometric mean of tyrosine 3-monooxygenase/tryptophan 5-monooxygenase activation protein Zeta (YWHAZ), β-actin (ACTB) and TUBB was used as an internal reference control for normalisation and used to calculate the ΔΔCt value.

**Table 2:** List of primers used for qRT-PCR

Gene	Forward Primer	Reverse Primer
*YWHAZ*	ACTGGGTCTGGCCCTTAACT	GGGTATCCGATGTCCACAATGTC
*ACTB*	CATTGCGGTGGACGATGGA	AGATCAAGATCATTGCTCCTCCTG
*TUBB*	GGACCGCATCTCTGTGTACT	CTGCCCCAGACTGACCAAATA
*COL1A1*	CCACGCATGAGCGGACCCTAA	ATTGGTGGGATGTCTTCGTCTTGG
*COL1A2*	ACAAGGCATTCGTGGCGATA	ACCATGGTGACCAGCGATAC
*COL3A1*	GACCTGGAGAGCGAGGATTG	GTCCATCGAAGCCTCTGTGT
*COL4A1*	GCCAGCAAGGTGTTACAGGATT	AGAAGGACACTGTGGGTCATCTATT
*LAMB1*	CCGGAAAGGAAGACGGGAAG	CGCCAGGTCCTGCTGTTTCTAA
*LAMB2*	CAGGCAGAGTTGACACGGAA	AGCCAGCACGCTTAGCAGTAG
*MMP2*	CCATGAAGCCCTGTTCACCA	CTTCTTGTCGCGGTCGTAGT
*MMP9*	GGCGCTCATGTACCCTATGT	TTCAGGGCGAGGACCATAGA
*TIMP1*	GGAATGCACAGTGTTTCCCT	GCCCTTTTCAGAGCCTTGGA

## Statistical analysis

Data are expressed as median [range] unless otherwise stated. Statistical analysis was performed using GraphPad Prism 7.01 software programme. Statistical significance was evaluated using Friedman’s test (for paired analyses) or Kruskal-Wallis test with Dunn’s post-hoc test. A *P* value of less than 0.05 was considered to be statistically significant.

## Data Availability

The data that support the findings of this study are available from the corresponding author upon reasonable request.
